# Location of Tandem Repeats on Wheat Chromosome 5B and the Breakpoint on the 5BS Arm in Wheat Translocation T7BS.7BL-5BS Using Single-Copy FISH Analysis

**DOI:** 10.3390/plants11182394

**Published:** 2022-09-14

**Authors:** Wei Zhang, Zongxiang Tang, Jie Luo, Guangrong Li, Zujun Yang, Manyu Yang, Ennian Yang, Shulan Fu

**Affiliations:** 1 College of Agronomy, Sichuan Agricultural University, Chengdu 611130, China; 2 Center for Informational Biology, School of Life Science and Technology, University of Electronic Science and Technology of China, Chengdu 611731, China; 3 Crop Research Institute, Sichuan Academy of Agricultural Sciences, Chengdu 610066, China

**Keywords:** single-copy FISH, ND-FISH, tandem repeats, wheat translocation, breakpoint

## Abstract

Wheat (*Triticum aestivum* L.) is rich in tandem repeats, and this is helpful in studying its karyotypic evolution. Some tandem repeats have not been assembled into the wheat genome sequence. Alignment using the blastn tool in the B2DSC web server indicated that the genomic sequence of 5B chromosome (IWGSC RefSeq v2.1) does not contain the tandem repeat pTa-275, and the tandem repeat (GA)_26_ distributed throughout the whole 5B chromosome. The nondenaturing fluorescence in situ hybridization (ND-FISH) using the oligonucleotide (oligo) probes derived from pTa-275 and (GA)_26_ indicated that one signal band of pTa-275 and two signal bands of (GA)_26_ appeared on the 5B chromosome of Chinese Spring wheat, indicating the aggregative distribution patterns of the two kinds of tandem repeats. Single-copy FISH indicated that the clustering region of pTa-275 and the two clustering regions of (GA)_26_ were located in ~160–201 Mb, ~153–157 Mb, and ~201–234 Mb intervals, respectively. Using ND-FISH and single-copy FISH technologies, the translocation breakpoint on the 5BS portion of the translocation T7BS.7BL-5BS, which exists widely in north-western European wheat cultivars, was located in the region from 157,749,421 bp to 158,555,080 bp (~0.8 Mb), and this region mainly contains retrotransposons, and no gene was found. The clustering regions of two kinds of tandem repeats on wheat chromosome 5B were determined and this will be helpful to improve the future sequence assembly of this chromosome. The sequence characteristics of the translocation breakpoint on the translocation T7BS.7BL-5BS obtained in this study are helpful to understand the mechanism of wheat chromosome translocation.

## 1. Introduction

Tandem repeats are an important part of the repetitive DNA sequences of plant genomes, and it is well known that they play functional roles in chromosome organization and genome evolution [1]. Tandem repeats can disclose the genome variations at the cytological level, and this can be used as corroborative evidence in plant systematics [2]. A lot of tandem repeats were discovered from the genome of Triticeae [3,4,5,6]. Some of these tandem repeats have not been assembled into the wheat genome sequence [7]. The accurate genomic locations of tandem repeats in wheat chromosomes contribute to a better understanding of their function. These tandem repeats were often used as fluorescence in situ hybridization (FISH) probes to investigate the structural alterations of wheat chromosomes [8,9,10,11]. Among the structural variations of wheat chromosomes, translocations are the most frequent type [9,12]. Many types of translocations between wheat chromosomes have been observed [9,12], and the reciprocal translocations T5BL.5BS-7BL and T7BS.7BL-5BS are noteworthy [13]. As early as 1994, it was reported that many wheat cultivars in France have these translocations [14]. Among the 538 wheat lines from the UK, 66% of them contained translocations T5BL.5BS-7BL and T7BS.7BL-5BS [15]. In recent years, these translocations occurred in some wheat cultivars bred from Sichuan, China, the hometown of Chinese Spring (*Triticum aestivum* L.) [11]. Learning the sequence characteristics of the translocation breakpoints on translocation chromosomes benefits the understanding of the mechanisms of chromosome translocation. For example, according to the sequence characteristics of the breakpoints on some rearranged chromosomes in human cancer cells, it was found that the sister chromatid breakage–fusion–bridge cycles were involved in chromosome structure variations [16] and repetitive DNA such as *Alu* elements can facilitate chromosome rearrangement [17]. The breakpoint on 7B in the chromosome T5BL.5BS-7BL was determined within about 5-kb of the region with GAA microsatellite [15]. However, the breakpoint on chromosome 5B was not determined because the chromosomes T7BS.7BL-5BS were derived from different translocation events [15]. A wheat cultivar Chuanmai 62 with the translocations T5BL.5BS-7BL and T7BS.7BL-5BS was derived from a French wheat line, and it is highly resistant to stripe rust [18]. In this study, the breakpoint on the 5BS arm in the translocation T7BS.7BL-5BS was investigated using tandem repeats and single-copy FISH analysis. Additionally, the positions of two kinds of tandem repeats on chromosome 5B of common wheat Chinese Spring were also determined using nondenaturing fluorescence in situ hybridization (ND-FISH) and single-copy FISH analyses.

## 2. Results

### 2.1. FISH Karyotype of Chromosomes 5B and 7B in Chinese Spring

**Figure 1:**
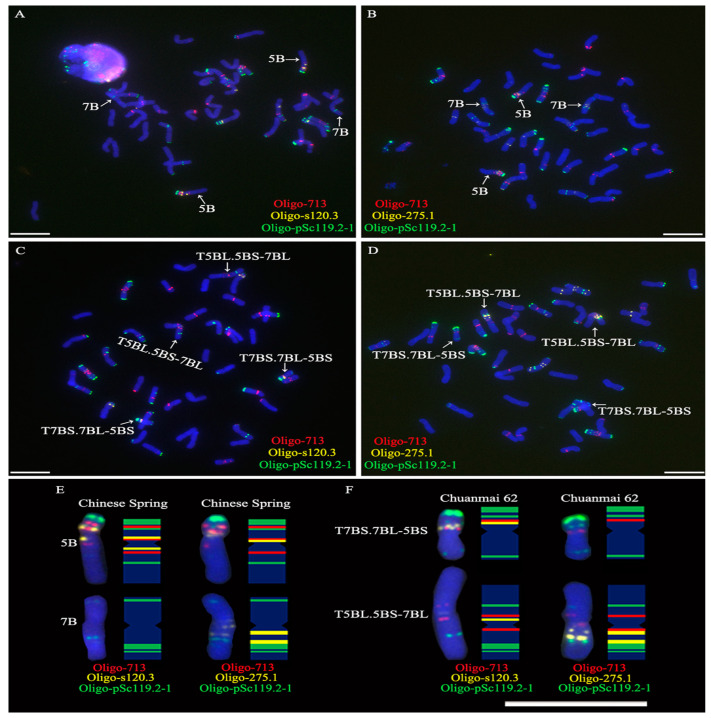
FISH analysis of root-tip metaphase chromosomes of Chinese Spring and Chuanmai 62 using oligo probes Oligo-713 (red), Oligo-s120.3 (yellow), Oligo-275.1 (yellow), and Oligo-pSc119.2-1 (green). (**A**,**B**) Cells of Chinese Spring. (**C**,**D**) Cells of Chuanmai 62. (**E**,**F**) Cut-and-paste chromosomes 5B of Chinese Spring and the translocations T5BL.5BS-7BL/T7BS.7BL-5BS of Chuanmai 62. The ideograms of these chromosomes are also exhibited. Scale bar: 10 μm and 50 μm.

Oligo probes Oligo-713, Oligo-s120.3, Oligo-275.1, and Oligo-pSc119.2-1 were used for ND-FISH analysis of the root-tip metaphase chromosomes of Chinese Spring. The probe Oligo-713 produced signals in the intercalary and pericentromeric regions of the short arm of chromosome 5B (5BS), and in the pericentromeric region of the long arm of chromosome 5B (5BL) (Figure 1A,B,E). The signals of Oligo-s120.3 could be observed on the pericentromeric regions of both 5BS and 5BL arms (Figure 1A,E). The signal of Oligo-275.1 appeared in the pericentromeric regions of the 5BS arm and the long arm of chromosome 7B (7BL), and in the proximally intercalary region of the 7BL arm (Figure 1B,E). The signals of Oligo-713 and Oligo-s120.3 in the pericentromeric region of the 5BS arm were almost overlapped (Figure 1A,E). No signals of Oligo-713 and Oligo-s120.3 were observed on the 7B chromosome (Figure 1A,B,E). Probe Oligo-pSc119.2-1 produced signals in the telomeric and intercalary regions of the 5BS arm, in the intercalary region of 5BL arms, in the distally intercalary region of the short arm of chromosome 7B (7BS) and the intercalary region of 7BL arm (Figure 1A,B,E). The FISH signal patterns of the four oligo probes on chromosomes 5B and 7B in this study were in accordance with that reported by Tang et al. [19].

### 2.2. ND-FISH Determining the Breakpoint Interval on 5BS Arm in T7BS.7BL-5BS

The four oligo probes were also used for ND-FISH analysis of the root-tip metaphase chromosomes of Chuanmai 62. The translocations T5BL.5BS-7BL and T7BS.7BL-5BS could be identified by the signal pattern of Oligo-pSc119.2-1 (Figure 1C,D,F). The signals of Oligo-713 and Oligo-s120.3 were observed in the pericentromeric region of the translocation T7BS.7BL-5BS (Figure 1C,F). It can be determined that this region was derived from the 5BS arm because the 7B chromosome does not contain the signals of the two probes. However, the signal of Oligo-275.1 in the pericentromeric region of the 5BS arm was not observed on translocation T7BS.7BL-5BS (Figure 1D,F). Therefore, it can be determined that the breakpoint interval on the 5BS arm was located between the pericentromeric signal sites of Oligo-713/Oligo-s120.3 and the pericentromeric signal site of Oligo-275.1. 

### 2.3. Single-Copy FISH Determining the Breakpoint Interval on the 5BS Arm in T7BS.7BL-5BS

**Figure 2:**
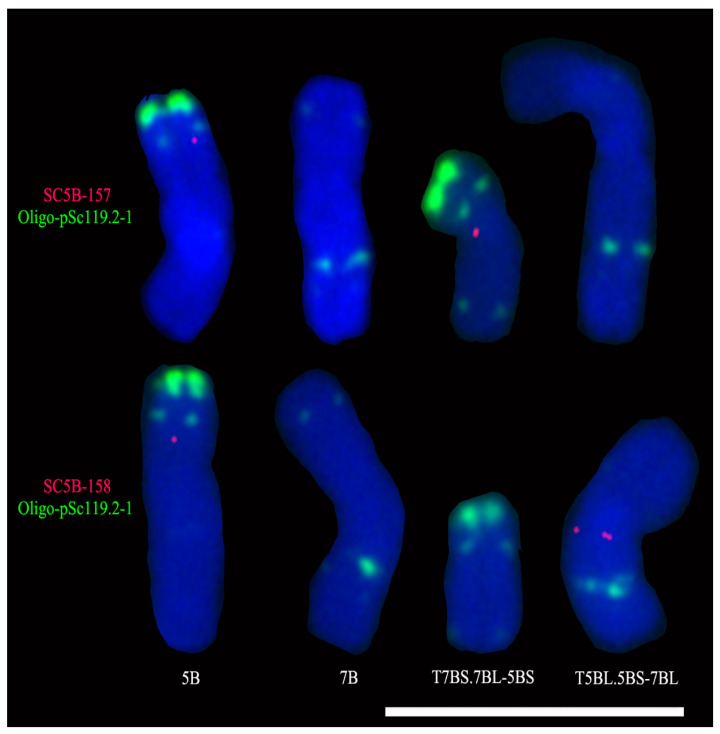
FISH analysis of chromosomes 5B and 7B of Chinese Spring and the translocations T5BL.5BS-7BL/T7BS.7BL-5BS of Chuanmai 62 using single-copy probes SC5B-157 (red) and SC5B-158 (red), and oligo probe Oligo-pSc119.2-1 (green). Cut-and-paste chromosomes are displayed. Scale bar: 100 μm.

The sequences of tandem repeats pTa-713, pTa-275, and (GA)_26_ (Oligo-s120.3) were used as queries to align against the genomic sequence of chromosome 5B of Chinese Spring (IWGSC RefSeq v2.1) using the blastn tool in B2DSC [7]. The alignment results indicated that tandem repeat pTa-713 clustered in three regions on chromosome 5B, and they are 87–88 Mb, 153–157 Mb, and 274–282 Mb intervals. The three aggregation regions of pTa-713 correspond to its FISH signal sites on chromosome 5B of Chinese Spring. The alignment did not find the aggregation regions of the sequence (GA)_26_ on chromosome 5B, and this is not in accordance with its FISH signal sites on this chromosome. In addition, the tandem repeat pTa-275 was not found in the genomic sequence of chromosome 5B of Chinese Spring (IWGSC RefSeq v2.1). Therefore, according to the results of ND-FISH and the sequence alignment, two single-copy sequences located in the 153–160 Mb interval of chromosome 5B were selected for designing primer pairs 157Mb-5B and 158Mb-5B (Table 1). The two primer pairs were used to amplify the single-copy sequences SC5B-157 and SC5B-158, respectively (Table 1). Subsequently, the single-copy FISH analysis of the root-tip metaphase chromosomes of Chinese Spring and Chuanmai 62 was carried out using the two sequences as probes. The signal of SC5B-157 was observed on chromosome 5B and the translocation T7BS.7BL-5BS but not on chromosome 7B and the translocation T5BL.5BS-7BL (Figure 2 and Figure S1A,C). The single-copy probe SC5B-158 produced a signal on chromosome 5B and the translocation T5BL.5BS-7BL but not on chromosome 7B and the translocation T7BS.7BL-5BS (Figure 2 and Figure S1B,D). So, it can be determined that the breakpoint interval of the 5BS arm in the translocation T7BS.7BL-5BS was between the signal sites of SC5B-157 and SC5B-158, corresponding to the ~0.8 Mb segment from 157,749,421 bp to 158,555,080 bp of chromosome 5B (Table 1). JBrowse visualization indicated that this region was rich in transposable elements and no gene existed in this region. The analysis using Giri Repbase also indicated that the ~0.8 Mb segment mainly contained *Gypsy* and *Copia* retrotransposons, and DNA transposons (Table S1). Therefore, the breakpoint region was mainly composed of repetitive DNA sequences.

### 2.4. Single-Copy FISH Determining the Position of Tandem Repeats on Chromosome 5B

**Figure 3:**
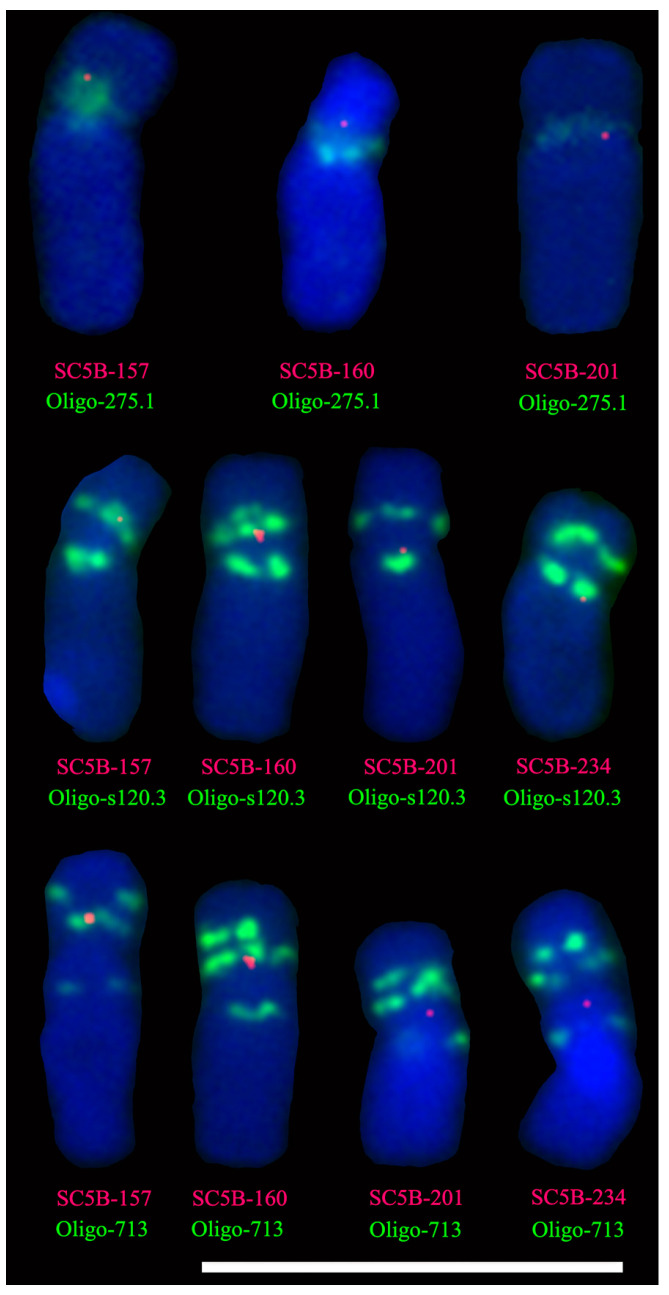
FISH analysis of chromosome 5B of wheat Chinese Spring using single-copy probes SC5B-157 (red), SC5B-160 (red), SC5B-158 (red), SC5B-201 (red), and SC5B-234 (red), and oligo probes Oligo-s120.3 (green) and Oligo-713 (green). Cut-and-paste chromosomes are displayed. Scale bar: 100 μm.

Additional three single-copy probes SC5B-160, SC5B-201, and SC5B-234 were also cloned (Table 1). Probes SC5B-157, SC5B-160, SC5B-201, and SC5B-234 were used to locate the clustering regions of tandem repeats pTa-275 and (GA)_26_ (Oligo-s120.3) on chromosome 5B of Chinese Spring. Both the signals of probes SC5B-157 and SC5B-160 were located on the distal side of the signal of Oligo-275.1, and the signal of SC5B-201 was near its proximal side (Figure 3 and Figure S2), hence, the clustering region of the tandem repeat pTa-275 was located at the ~160–201 Mb interval. On the 5BS arm, the signal of SC5B-157 overlapped with both of the pericentromeric signals of Oligo-713 and Oligo-s120.3, and the signal of SC5B-160 was located on the proximal sides of the signals of the two oligo probes (Figure 3, Figures S3 and S4). This confirmed that the pericentromeric signal of Oligo-713 on the 5BS arm overlapped with that of Oligo-s120.3. The alignment using the blastn tool in B2DSC indicated that one of the clustering regions of pTa-713 on chromosome 5B was the 153–157 Mb interval, and the signal of SC5B-157 overlapped with both of the pericentromeric signals of Oligo-713 and Oligo-s120.3 on the 5BS arm. So, the clustering region of the tandem repeat (GA)_26_ on the 5BS arm should be at the ~153–157 Mb interval, as the pTa-713. The pericentromeric signal of Oligo-s120.3 on the 5BL arm was located between the two signal sites of SC5B-201 and SC5B-234, indicating that the clustering region of (GA)_26_ on the 5BL arm was the ~201–234 Mb interval (Figure 3 and Figure S3C,D).

## 3. Discussion

### 3.1. Methods for the Determination of Breakpoint in Wheat Translocation Chromosome

It was reported that the breakpoint in translocation chromosome T5BS-7BL.7BS was located in the region distal to the pericentromeric C-band on the 5BS arm [12]. This breakpoint region was determined between 99 Mb and 151 Mb using oligo probes derived from tandem repeats [7]. The genomic sequencing method did not determine the breakpoint in the 5BS arm in the translocation T5BS-7BL.7BS because of the different translocation events on 5BS/7BS [15]. In this study, a difference in the signal site of Oligo-713 in the intercalary region on the 5BS arm was observed between Chinese Spring and Chuanmai 62 (Figure 1). The polymorphism of this signal site among chromosome 5B has already been reported [11]. Whereas, the pericentromeric signal sites of Oligo-713 and the signal sites of Oligo-s120.3 and Oligo-275.1 on the 5BS arms are conserved [11]. Hence, according to the signal patterns of Oligo-713, Oligo-s120.3, and Oligo-275.1, the breakpoint in the 5BS arm of the translocation T5BS-7BL.7BS could be roughly determined to be located between the pericentromeric signal site of Oligo-275.1 and the pericentromeric signal sites of Oligo-713/Oligo-s120.3. Then, combined with the genomic sequence of chromosome 5B, the breakpoint interval between 157,749,421 bp and 158,555,080 bp (~0.8 Mb segment) was determined using the single-copy FISH analysis. The breakpoint in the translocation 4AL/5AL of Triticeae was located in an ~1000 bp region with genes [20]. Subsequently, this breakpoint was narrowed down to a 125 bp interval using DNA markers [21]. Using DNA markers, the recombination breakpoint in translocation 5DS-5BS was located in a 39 bp conserved region within a predicted gene [22]. The results in these previous studies indicated that relatively precise positions of the breakpoints in wheat translocations could be determined using molecular markers. However, molecular markers were used to determine the breakpoints on the 3AL arm in the wheat-*Thinopyrum intermedium* translocation T3AL-7J^S^S.7J^S^L, and the breakpoint interval was about 7 Mb [23]. The breakpoints in translocations 4AL/5AL and 5DS-5BS exist in the regions with genes [20,22]. It is easy to obtain high-density and chromosome-specific markers in these regions. Perhaps, for the breakpoint region on the 3AL arm in the translocation T3AL-7J^S^S.7J^S^L, this condition did not exist. BAC clones were used as probes for the FISH analysis of ring minichromosomes derived from *Arabidopsis*, and two clones could not be used to determine the breakpoint of minichromosomes due to the abundance of repetitive DNA sequences in the two clones [24]. Similarly, in the present study, the breakpoint interval on the 5BS arm was mainly composed of repetitive DNA sequences. Using the sequence of the ~0.8 Mb segment, we failed to design 5BS-specific markers and to find more single-copy sequences, therefore, it is difficult to narrow down this kind of breakpoint. In spite of this, the ND-FISH and single-copy FISH analyses combined with a genomic sequence are still useful in the investigation of the breakpoint of wheat translocation. The results in this study and previous studies also displayed the complexity of breakpoints in wheat translocations.

### 3.2. Determination of the Position of Tandem Repeats on Wheat Chromosome

The genome of wheat is rich in tandem repeats [5,6,7]. However, some tandem repeats were not assembled into the genomic sequence of Chinese Spring [7]. In this study, the tandem repeat pTa-275 was not found in the genomic sequence of chromosome 5B (IWGSC RefSeq v2.1), and the distribution pattern of the tandem repeat Oligo-s120.3 in the sequence of chromosome 5B (IWGSC RefSeq v2.1) was not in accordance with its FISH signal pattern on this chromosome. All these results indicated that the assembly of the wheat genomic sequence needs to be improved. Learning the accurate distribution patterns of tandem repeats in the wheat genome contributes to a better understanding of their function in gene regulation [7]. The FISH analysis using tandem repeats as probes is helpful in the assembly of chromosome-based genome sequences [25]. The single-copy FISH was used to investigate chromosome rearrangements and collinearity of homoeologous in closely related species [26,27,28,29,30]. Single-copy FISH was also used to determine the recombination interval on chromosome 5A [31] and the position of centromeres on rye chromosomes [32]. In this study, the clustering regions of tandem repeats Oligo-s120.3 and pTa-275 on chromosome 5B were determined by the ND-FISH using oligo probes based on tandem repeats and the single-copy FISH. Therefore, FISH technology is important for improving the assembly of genome sequences [7,25].

## 4. Materials and Methods

### 4.1. Plant Materials

Common wheat Chinese Spring (*Triticum aestivum* L.) and wheat cultivar Chuanmai 62 (*Triticum aestivum* L.) were used in this study. Cultivar Chuanmai 62 contains reciprocal translocations T5BL.5BS-7BL/T7BS.7BL-5BS [11,18].

### 4.2. Nondenaturing Fluorescence in situ Hybridization (ND-FISH) Analysis

Oligonucleotide (oligo) probes Oligo-pSc119.2-1 [33,34], Oligo-s120.3, Oligo-275.1, and Oligo-713 [5,19] were used for ND-FISH analysis. The nucleotide sequence of Oligo-s120.3 is microsatellite DNA (GA)_26_ [19]. The procedure of ND-FISH was carried out according to the methods described by Fu et al. [35]. 

### 4.3. Single-Copy FISH Analysis

The blastn tool in the B2DSC web server (http://mcgb.uestc.edu.cn/b2dsc, accessed on 30 March 2022) [7] was used to align the tandem repeats pTa-713, pTa-275.1 [5] and Oligo-s120.3 [5,19] with the full-length sequence of chromosome 5B of Chinese Spring (IWGSC RefSeq V2.1). Some single-copy sequences near the clustering position of pTa-713 were selected as probes for single-copy FISH analysis. Four primer pairs were designed to amplify the single-copy probes (Table S1). The obtaining of single-copy sequences and the single-copy FISH analysis were carried out according to the methods described by Zou et al. [31].

### 4.4. The Analysis of the Characters of the Sequence in the Breakpoint Region in 5BS Arm

When the region containing the breakpoint in the 5BS arm was determined, the Giri Repbase (https://www.girinst.org/censor/index.php; accessed on 14 June 2022) [36] and the JBrowse in Triticeae Multi-omics Center (http://202.194.139.32/jbrowse.html; accessed on 29 March 2022) was used to analyze the sequence in this region.

## 5. Conclusions

The breakpoint interval on the 5BS arm of the translocation T5BS-7BL.7BS was rapidly determined using ND-FISH and single-copy FISH technologies, and they are useful in determining the breakpoint regions with repetitive DNA sequences in wheat translocations. The sequence characteristics of the translocation breakpoint region on the T7BS.7BL-5BS translocation were learned and this is useful to understand the mechanism of wheat chromosome translocation. Additionally, the clustering regions of tandem repeats in chromosomes can be determined using these methods and the results contribute to the future perfect assembly of the wheat genomes.

## Figures and Tables

**Table 1:** The information of the single-copy probes used in this study.

Name of Single-Copy Probe	Name of Primers Used to Amplify Single-Copy Sequence	Nucleotide Sequence of Primer (5′-3′)	The Position on the Chromosome 5B of Chinese Spring (IWGSC RefSeq V2.1) (bp)
SC5B-157	157Mb-5BF	ACCCGCCAGAATCATCACTG	157,749,421–157,752,276
	157Mb-5BR	GGGAATCCAAGCCACGATCT	
SC5B-158	158Mb-5BF	TTCCAATTCCAGACCCTGCC	158,552,855–158,555,080
	158Mb-5BR	CTGTTCTTGCTATTCCGCGC	
SC5B-160	160Mb-5BF	ACTCGGTGGTGCATGAAACT	160,001,176–160,003,706
	160Mb-5BR	TGGACTAGTCGAGGGGTCTG	
SC5B-201	201Mb-5BF	TCCCTCACTGGTACCATCCC	201,713,962–201,711,028
	201Mb-5BR	CCTGGTATGCAGGAATCCCC	
SC5B-234	234Mb-5BF	AAAACCATTGCCACAGGTGC	234,929,992–234,932,621
	234Mb-5BR	ATTCGGTCGATCCTACACGC	

## Data Availability

The materials used in this study are available on request from the corresponding author. The sequences of primers used in this study can be obtained in Table 1.

## Supplementary Materials

The following supporting information can be downloaded at: https://www.mdpi.com/article/10.3390/plants11182394/s1, Figure S1: FISH analysis using single-copy probes SC5B-157 (red) and SC5B-158 (red), and oligo probe Oligo-pSc119.2-1 (green); Figure S2: FISH analysis using single-copy probes SC5B-157 (red), SC5B-160 (red), and SC5B-201 (red), and oligo probe Oligo-275.1 (green); Figure S3: FISH analysis using single-copy probes SC5B-157 (red), SC5B-160 (red), SC5B-201 (red), and SC5B-234 (red), and oligo probe Oligo-s120.3 (green); Figure S4: FISH analysis using single-copy probes SC5B-157 (red), SC5B-160 (red), SC5B-201 (red), and SC5B-234 (red), and oligo probe Oligo-713 (green); Table S1: Characters of the 0.8 Mb sequence with breakpoint in the 5BS arm of T7BS.7BL-5BS.
