# Supplementary material for: Location of Tandem Repeats on Wheat Chromosome 5B and the Breakpoint on the 5BS Arm in Wheat Translocation T7BS.7BL-5BS Using Single-Copy FISH Analysis
Source: Plants (Basel). 2022 Sep 14;11(18):2394. doi: 10.3390/plants11182394 (PMC9502598; doi:10.3390/plants11182394)
Supplement: Supplementary file 1 [file plants-11-02394-s001.zip › plants-1831664-supplementary.pdf]

SUPPLEMENTARY MATERIAL

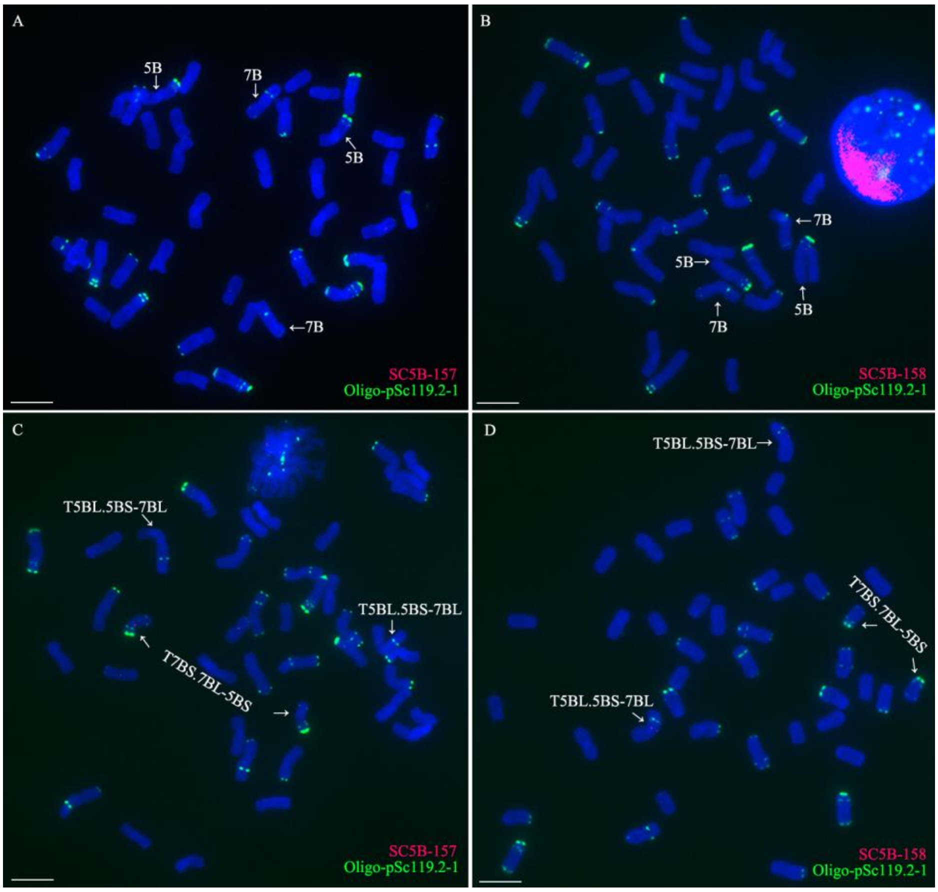

Figure S1. FISH analysis using single-copy probes SC5B-157 (red) and SC5B-158 (red), and oligo probe Oligo-pSc119.2-1 (green). (A, B) The root-tip metaphase chromosomes of Chinese Spring. (C, D) The root-tip metaphase chromosomes of Chuanmai 62. Scale bar is 10 μm.

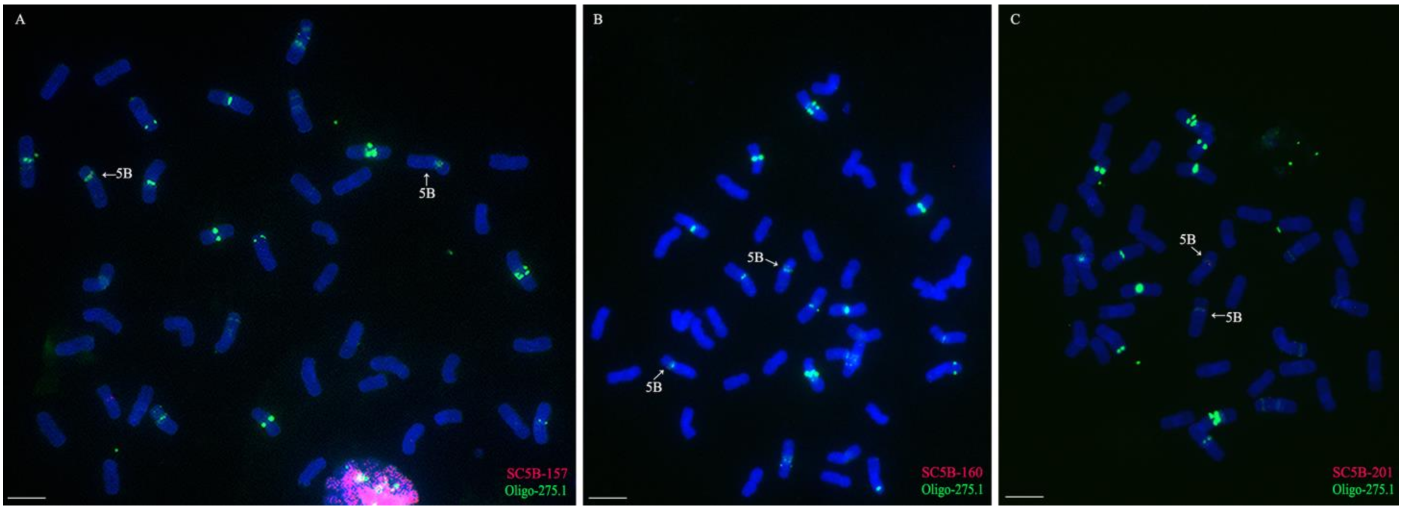

Figure S2. FISH analysis using single-copy probes SC5B-157 (red), SC5B-160 (red) and SC5B-201 (red), and oligo probe Oligo-275.1 (green). (A, B, C) Three root-tip metaphase cells of Chinese Spring. Scale bar is 10 μm.

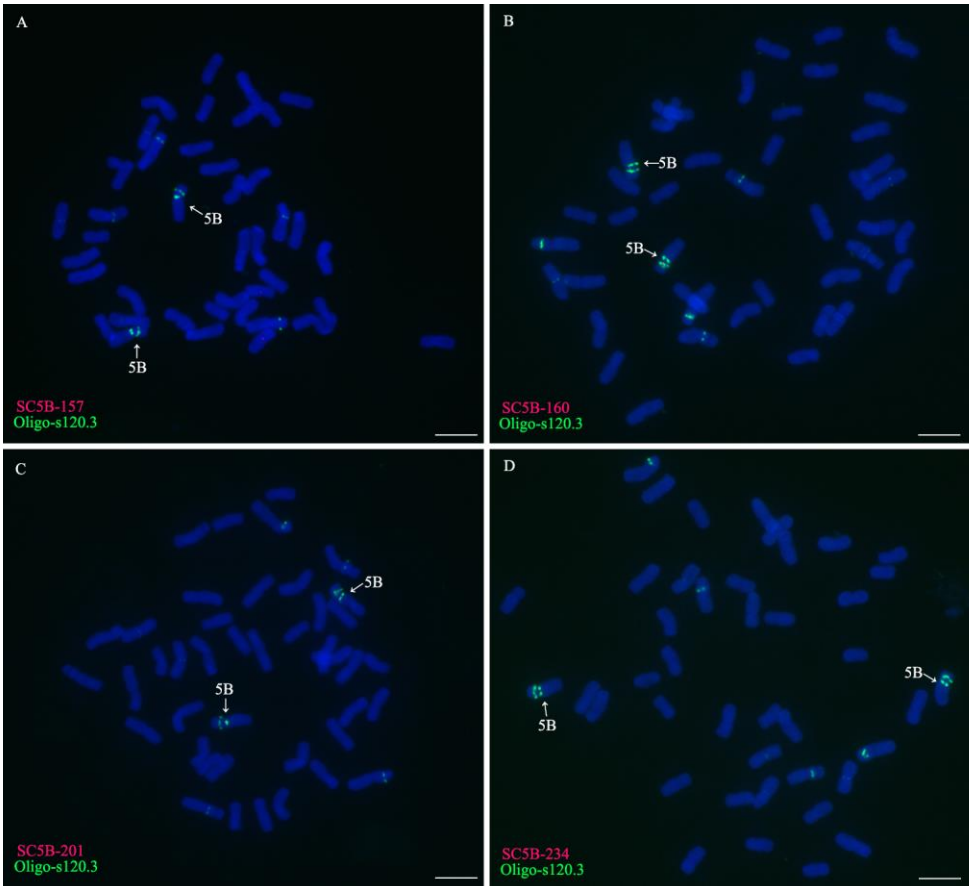

Figure S3. FISH analysis using single-copy probes SC5B-157 (red), SC5B-160 (red), SC5B-201 (red) and SC5B-234 (red), and oligo probe Oligo-s120.3 (green). (A, B, C, D) Four root-tip metaphase cells of Chinese Spring. Scale bar is 10 μm.

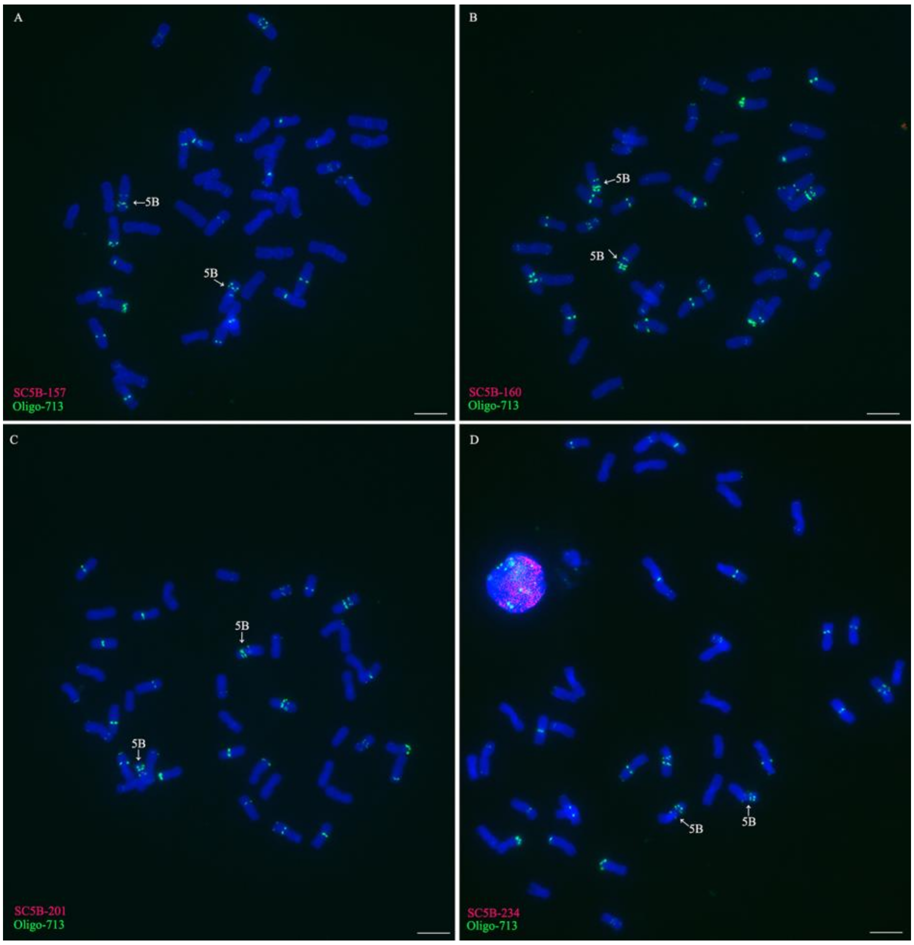

Figure S4. FISH analysis using single-copy probes SC5B-157 (red), SC5B-160 (red), SC5B-201 (red) and SC5B-234 (red), and oligo probe Oligo-713 (green). (A, B, C, D) Four root-tip metaphase cells of Chinese Spring. Scale bar is 10 μm.

Table S1. Characters of the 0.8 Mb sequence with breakpoint in the 5BS arm of T7BS.7BL-5BS.

Type of sequence	Position in the 0.8 Mb sequence (bp)		Position in the genomic sequence of 5B chromosome (IWGSC RefSeq v2.1) (bp)	
	Stard	End	Strard	End
LTR/Gypsy	7	770	157749428	157750191
LTR/Gypsy	2961	3028	157752382	157752449
LTR	3060	4626	157752481	157754047
LTR/Gypsy	5179	5503	157754600	157754924
LTR	5506	7502	157754927	157756923
LTR/Gypsy	7512	16360	157756933	157765781
LTR	17002	18554	157766423	157767975
LTR/Gypsy	22537	37034	157771958	157786455
LTR/Copia	37035	47147	157786456	157796568
LTR/Gypsy	47222	64049	157796643	157813470
LTR	64084	65659	157813505	157815080
LTR/Gypsy	65660	95783	157815081	157845204
DNA/En-Spm/CACTA	95790	101520	157845211	157850941
LTR/Gypsy	101521	101741	157850942	157851162
DNA/En-Spm/CACTA	101745	103501	157851166	157852922
LTR/Gypsy	103502	106519	157852923	157855940

DNA/En-Spm/CACTA	106520	109959	157855941	157859380
LTR/Gypsy	109960	120069	157859381	157869490
DNA	123171	123436	157872592	157872857
DNA/En-Spm/CACTA	123795	123930	157873216	157873351
LTR	124304	124569	157873725	157873990
DNA/En-Spm/CACTA	124835	124998	157874256	157874419
LTR/Copia	125093	139761	157874514	157889182
DNA/En-Spm/CACTA	140162	143023	157889583	157892444
LTR	143241	145496	157892662	157894917
DNA/En-Spm/CACTA	145501	153145	157894922	157902566
DNA	153931	161973	157903352	157911394
DNA/En-Spm/CACTA	162022	162175	157911443	157911596
LTR/Gypsy	166917	167879	157916338	157917300
LTR	168121	176475	157917542	157925896
LTR/Gypsy	176944	178590	157926365	157928011
LTR	178672	185696	157928093	157935117
LTR/Gypsy	185699	187031	157935120	157936452
LTR/Copia	187032	189475	157936453	157938896
LTR	189476	191226	157938897	157940647
LTR/Copia	191227	193915	157940648	157943336
LTR	193916	195667	157943337	157945088
LTR/Copia	195668	199501	157945089	157948922
LTR/Gypsy	199509	224883	157948930	157974304
LTR/Copia	224884	226183	157974305	157975604
LTR	226184	227930	157975605	157977351
LTR/Copia	227931	230926	157977352	157980347
LTR/Gypsy	230927	241792	157980348	157991213
LTR/Copia	242196	244327	157991617	157993748
LTR	244328	245984	157993749	157995405
LTR/Copia	245985	253267	157995406	158002688
LTR	253269	254988	158002690	158004409
LTR/Copia	254990	261164	158004411	158010585
LTR	261165	262746	158010586	158012167
LTR/Copia	262787	264484	158012208	158013905
LTR	264485	266235	158013906	158015656
LTR/Copia	266236	278990	158015657	158028411
LTR	278992	280737	158028413	158030158
LTR/Copia	280738	290955	158030159	158040376
LTR	290956	292703	158040377	158042124
LTR/Copia	292704	297720	158042125	158047141
LTR/Gypsy	297755	308809	158047176	158058230
LTR/Copia	308810	308897	158058231	158058318
LTR	308898	310614	158058319	158060035
LTR/Copia	310616	314533	158060037	158063954
LTR	314534	316278	158063955	158065699
LTR/Copia	316279	321377	158065700	158070798
LTR	321378	323126	158070799	158072547

LTR/Copia	323127	328231	158072548	158077652
LTR	328232	330918	158077653	158080339
LTR/Copia	330919	332059	158080340	158081480
LTR	332060	333810	158081481	158083231
LTR/Copia	333811	338652	158083232	158088073
LTR	338653	342129	158088074	158091550
LTR/Copia	342130	347253	158091551	158096674
LTR	347254	349000	158096675	158098421
LTR/Copia	349001	354129	158098422	158103550
LTR	354130	355490	158103551	158104911
LTR/Copia	355492	358280	158104913	158107701
LTR	358281	361638	158107702	158111059
LTR/Copia	361639	362786	158111060	158112207
LTR	362787	364511	158112208	158113932
LTR/Copia	364542	369656	158113963	158119077
LTR	369657	371403	158119078	158120824
LTR/Copia	371404	376494	158120825	158125915
LTR	376495	377247	158125916	158126668
LTR/Copia	377282	382934	158126703	158132355
LTR	382935	384650	158132356	158134071
LTR/Copia	384652	389954	158134073	158139375
LTR	389955	391676	158139376	158141097
LTR/Copia	391677	396758	158141098	158146179
LTR	396759	397700	158146180	158147121
LTR/Gypsy	398140	410260	158147561	158159681
LTR	410262	411453	158159683	158160874
LTR/Copia	411454	416557	158160875	158165978
LTR	416558	418293	158165979	158167714
LTR/Copia	418294	423428	158167715	158172849
LTR	423429	426483	158172850	158175904
LTR/Copia	426491	428827	158175912	158178248
LTR	428828	430547	158178249	158179968
LTR/Copia	430548	434069	158179969	158183490
LTR	434070	435783	158183491	158185204
LTR/Copia	435785	441086	158185206	158190507
LTR	441087	442801	158190508	158192222
LTR/Copia	442803	449526	158192224	158198947
LTR	449536	451236	158198957	158200657
LTR/Copia	451237	459562	158200658	158208983
LTR	459597	461314	158209018	158210735
LTR/Copia	461315	466615	158210736	158216036
LTR	466617	467855	158216038	158217276
LTR/Copia	467864	470366	158217285	158219787
LTR	470367	472115	158219788	158221536
LTR/Copia	472116	484118	158221537	158233539
LTR	484119	485848	158233540	158235269
LTR/Copia	485849	486787	158235270	158236208
LTR/Gypsy	486788	487966	158236209	158237387
DNA/En-Spm/CACTA	488124	488770	158237545	158238191
LTR/Gypsy	488943	494260	158238364	158243681
DNA/En-Spm/CACTA	494417	495062	158243838	158244483

LTR/Gypsy	495235	496541	158244656	158245962
LTR/Copia	496542	504167	158245963	158253588
LTR	504168	505920	158253589	158255341
LTR/Copia	505921	510879	158255342	158260300
LTR	510880	512626	158260301	158262047
LTR/Copia	512628	517816	158262049	158267237
LTR	517817	519538	158267238	158268959
LTR/Copia	519579	521275	158269000	158270696
LTR	521281	522989	158270702	158272410
LTR/Copia	522991	531308	158272412	158280729
LTR	531309	533053	158280730	158282474
LTR/Copia	533054	539448	158282475	158288869
LTR	539454	541155	158288875	158290576
LTR/Copia	541156	546457	158290577	158295878
LTR	546461	548169	158295882	158297590
LTR/Copia	548170	553802	158297591	158303223
LTR	553804	554786	158303225	158304207
LTR/Copia	554787	559991	158304208	158309412
LTR	559992	561717	158309413	158311138
LTR/Copia	561718	570685	158311139	158320106
LTR	570686	572434	158320107	158321855
LTR/Copia	572435	577521	158321856	158326942
LTR	577522	579122	158326943	158328543
LTR/Gypsy	579123	593234	158328544	158342655
LTR/Copia	593235	601089	158342656	158350510
LTR	601090	602836	158350511	158352257
LTR/Copia	602837	607966	158352258	158357387
LTR	607968	610616	158357389	158360037
LTR/Copia	610618	611771	158360039	158361192
LTR	611772	613521	158361193	158362942
LTR/Copia	613522	618623	158362943	158368044
LTR	618624	620344	158368045	158369765
LTR/Copia	620346	625610	158369767	158375031
LTR	625611	629066	158375032	158378487
LTR/Copia	629067	634189	158378488	158383610
LTR	634190	635941	158383611	158385362
LTR/Copia	635942	641073	158385363	158390494
LTR	641074	642823	158390495	158392244
LTR/Copia	642824	647916	158392245	158397337
LTR	647917	649669	158397338	158399090
LTR/Copia	649671	654972	158399092	158404393
LTR	654973	657680	158404394	158407101
LTR/Copia	657687	659338	158407108	158408759
LTR	659339	661088	158408760	158410509
LTR/Copia	661089	666076	158410510	158415497
LTR	666077	666246	158415498	158415667
LTR/Copia	666247	674802	158415668	158424223
LTR	674803	676552	158424224	158425973
LTR/Copia	676553	677181	158425974	158426602
DNA/MuDR	677182	677685	158426603	158427106
LTR/Copia	677686	682184	158427107	158431605
LTR	682185	683859	158431606	158433280
LTR/Copia	683860	688974	158433281	158438395

LTR	688975	690720	158438396	158440141
LTR/Copia	690722	700634	158440143	158450055
LTR	700635	702381	158450056	158451802
LTR/Copia	702387	707504	158451808	158456925
LTR	707505	709240	158456926	158458661
LTR/Copia	709251	715487	158458672	158464908
LTR	715489	717211	158464910	158466632
LTR/Copia	717215	719551	158466636	158468972
LTR	719559	721304	158468980	158470725
LTR/Copia	721305	726397	158470726	158475818
LTR	726398	729446	158475819	158478867
LTR/Copia	729447	734533	158478868	158483954
LTR	734535	738027	158483956	158487448
LTR/Copia	738028	742105	158487449	158491526
LTR	742107	750370	158491528	158499791
LTR/Gypsy	750373	760484	158499794	158509905
LTR	760485	762059	158509906	158511480
LTR/Gypsy	762060	766618	158511481	158516039
DNA	766737	766819	158516158	158516240
LTR	767532	774175	158516953	158523596
DNA/En-Spm/CACTA	776123	777034	158525544	158526455
LTR/Gypsy	777045	781743	158526466	158531164
LTR	781806	785451	158531227	158534872
DNA/En-Spm/CACTA	785452	788064	158534873	158537485
LTR/Copia	788065	788750	158537486	158538171
DNA	789011	789078	158538432	158538499
LTR/Copia	789536	799233	158538957	158548654
DNA	799476	799543	158548897	158548964
DNA/En-Spm/CACTA	800004	800226	158549425	158549647
NonLTR/L1	802963	803194	158552384	158552615
DNA/En-Spm/CACTA	803669	803868	158553090	158553289
